# Twinning in Holstein-Friesian Dairy Cows: Proportion Carried to Term and Calf Sex Ratios 

**DOI:** 10.3390/vetsci2030131

**Published:** 2015-07-07

**Authors:** Peter D. Cockcroft, Emma J. Sorrell

**Affiliations:** 1 School of Animal and Veterinary Sciences, University of Adelaide, Roseworthy Campus, Roseworthy, SA 5371, Australia; 2 The Brambles Veterinary Surgery, Churchdown, GL3 2HE, UK; E-Mail: info@brambles-vets.co.uk

**Keywords:** dairy, cattle, twinning, sex ratio, abortion

## Abstract

The purpose of this study was to investigate the proportion of twins carried to term and the sex ratio of twin calves at birth in Holstein-Friesian dairy cattle kept on commercial farms in Devon and Cornwall, England. Ten farms were used in the study. Fifty four cows with twin pregnancies were identified using trans-rectal ultra-sonographic examination between 30 and 70 days of gestation. The farm records were subsequently used to derive the number of calves born. Farm records of 66 additional sets of twin births with the sex of the calves recorded were also identified. Of the 54 cows diagnosed with twin pregnancies, 16 cows (29.6%) aborted or absorbed both fetuses, 11 cows (20.4%) carried one calf to term and 27 cows (50%) carried both calves to term. In the calf sex analysis of the additional 66 sets of twins: 13♂♂ calves (19.7%), 18 ♀♀ calves (27.3%) and 35 ♂♀ calves (53.0%). There was no statistically significant difference from an expected ratio of 1♂♂:2♂♀:1♀♀ (*p* = 0.61). This study provides bench marks for the expected abortion/absorption rates following the early ultra-sonographic diagnosis of twin pregnancies in comparable populations and supports earlier observations that the expected  sex ratio for twinning approximates to1♂♂:2♂♀:1♀♀.

## 1. Introduction 

Twinning in dairy cattle is associated with higher milk yields [1], an increased incidence of abortion [1], dystocia [1], retained fetal membranes [1], metritis [1], Freemartinism [1,2] and an extended calving to conception interval [1,3]. Over 95% of female calves twinned to a male are sterile [4]. The additional cost of twinning has been reviewed [3] and estimated at £109 [5]. The incidence of twinning reported in dairy herds varies between studies, herds and parities but is an average of 4% has been reported in UK herds [5]. The rate of single and double embryonic/fetal losses in twin pregnancies in Holstein cows have been published [6,7]. Large scale studies of the twin calf sex ratios have been reported [8,9,10].

The purpose of this study was to investigate the proportion of twins carried to term and the calf sex ratio of twin calves in Holstein-Friesian dairy cattle kept on 10 commercial farms in Devon and Cornwall, England.

## 2. Experimental Section 

Ten Friesian-Holstein dairy farms in Devon and Cornwall were used in this study. Herd size ranged from 70 to 310 animals with a mean of 168. None of the farms instigated a specifically designed management protocol to deal with cows identified as carrying twins. Fifty four cows with twin pregnancies were identified during routine trans-rectal ultra-sonographic examination for pregnancy. A 7.5 MHz linear probe was used. The operator was a large animal veterinarian experienced in ultra-sonographic pregnancy diagnosis. Twins were identified between 30 and 70 days of gestation according to the last recorded service date and a diagnosis of twinning was made on the observation of two embryos/fetuses. The farm records were subsequently used to derive the number of calves born following the ultra-sonographic diagnosis. 

Farm records of an additional 66 sets of twin births with the sex of the calves recorded were identified. A chi-squared test was used to analyse the data for a random distribution giving a gender ratio of 1♂♂:2♂♀:1♀♀.

## 3. Results

Of the 54 cows diagnosed with twin pregnancies between 30 and 70 days of gestation, 16 cows (29.6%) aborted or reabsorbed both fetuses, 11 cows (20.4%) carried one calf to term and 27 cows (50%) carried both to term. In the calf sex ratio analysis of the additional 66 sets of twins there were 13♂♂ calves (19.7%), 18 ♀♀ calves (27.3%) and 35 ♂♀ calves (53.0%). There was no statistically significant difference when the numbers of calves within each category were compared to an expected ratio of 1♂♂:2♂♀:1♀♀ (*p* = 0.61). 

## 4. Discussion

A comparative study of gestation losses in beef cattle at the US Meat Animal Research Centre found that 12.2% of cows with twin pregnancies aborted or reabsorbed both fetuses and 4.6% aborted or reabsorbed one fetus [11]. López-Gatius and Hunter (2005) [6] reported embryonic/fetal losses in 211 twin pregnancies in high yielding dairy cows in Spain. Loss of both pregnancies occurred in 73 cows (24.2%), with 13 cows (6.2%) maintaining a single pregnancy. Silva-del-Río *et al*. (2009) [7] reported outcomes for 98 twin pregnancies in Holstein cows in the USA where 13 cows (13.3%) lost both embryos/fetuses, and 11 cows (11.2%) lost a single embryo/fetus. The abortion/absorbtion rate in this study were higher and may be related to management or health status differences between the populations.

A sex ratio of 1♂♂:2♂♀:1♀♀ was reported in a large population of Holstein cows [12]. The calf sex ratio amongst 925 sets of twins in dairy cows was 24.9% ♂♂:48.7% ♂♀:26.4%♀♀ in the USA study [8]. An analysis of 96,069 twin births found a twin sex ratio of 30.1% ♂♂:43.6%♂♀:26.3%♀♀ in USA dairy cows [9]. Ghavi Hossein-Zadeh *et al.* (2008) reported a calf sex ratio in 4,045 twin births in Holstein cows in Iran of 28.2%♂♂:48.8%♂♀:22.9%♀♀ [10].

Within the power of this study the distribution of the sexes of the twins were consistent with this random distribution of the sexes. In a review of the literature of twinning in cattle 92.6% were classified as fraternal or dizygotic twins and 7.4% classified as monozygotic or identical twins [13,14]. Therefore the assumption of a random distribution of the sexes will be approximate rather than absolute.

## 5. Conclusion

This study provides bench marks for the expected abortion/absorption rates following the early ultra-sonographic diagnosis of twin pregnancies in comparable populations and supports earlier observations that the expected sex ratio for twinning approximates to 1♂♂:2♂♀:1♀♀.
